# Outcome of Gartland type II and type III supracondylar fractures treated by Blount's technique

**DOI:** 10.4103/0019-5413.58612

**Published:** 2010

**Authors:** Antoine de Gheldere, Damien Bellan

**Affiliations:** Department of Paediatric Orthopaedic Surgery, CHU Brabois, Children's Hospital, Rue du Morvan, F-54500, Vandoeuvre les Nancy, France

**Keywords:** Blount's technique, supracondylar fractures, closed reduction and immobilization

## Abstract

**Background:**

According to some orthopedic surgeons, almost all supracondylar humerus fractures should be treated operatively by reduction and pinning. While according to others, closed reduction and immobolization should be used for Gartland type II and some type III fractures. However, the limit of this technique remains unclear. We present 74 patients with displaced extension-type supracondylar fractures treated by closed reduction and immobilization with a collar sling fixed to a cast around the wrist. The purpose of the study is to give a more precise limitation of this technique.

**Materials and Methods:**

Retrospective data acquisition of 74 patients with a Gartland type II or type III fractures treated by closed reduction and immobilization (Blount's technique) between January 2004 and December 2007 was done. The mean age was 6.3 years (range, 2–11). The mean time of follow-up was 6.5 months (range, 3–25). All open injuries and complex elbow fracture dislocations or T-condylar fractures were excluded from the study. All patients were evaluated with standardized anteroposterior and true lateral x-rays of the elbow, and Flynn criteria were used for functional assessment.

**Results:**

Gartland type II fractures had 94% good or excellent final results. Gartland type III fractures had 73% good or excellent final result. The Gartland type III outcome depended on the displacement. The fractures remained stable in 88% for the posterior displacement, and 58% for the posteromedial displacement. These displacements were mild. However, for the posterolaterally displaced fractures, only 36% were stable; 36% had a mild displacement and 27% had a major displacement.

**Conclusion:**

Pure posterior displacement is more stable than posteromedial displacement which is more stable than posterolaterally displaced fractures. This study suggests that Gartland type II and pure posterior or posteromedial displaced Gartland type III fractures can be treated by closed reduction and immobilization with success.

## INTRODUCTION

Supracondylar humeral fractures (SHF) in children are the most common injuries of the elbow.^1^,^2^ The current literature on SHF suggests that percutaneous pinning should be used for most of the extension-type fractures, even for the minimally displaced ones.^2^,^3^ According to some authors, closed reduction and immobilization is associated with a significant percentage of early and late complications, including Volkmann ischemic contracture and cubitus varus.^4^–^8^

Blount's technique (closed reduction and immobilization)^9^ is used in our institution for displaced extension-type SHF. This study was undertaken to report our results and to compare the outcome of Gartland type II and type III fractures. Furthermore, we tried to define the fractures that can be managed by this technique and the ones that should be treated by surgical fixation.

## MATERIALS AND METHODS

Between 2004 and 2007, 234 displaced extension supracondylar fractures of the humerus in children were treated. All open injuries, complex elbow fracture dislocations or T-condylar fractures were excluded from the study. We retrospectively reviewed medical records and radiographs of 77 consecutive displaced fractures treated in our institution by Blount's technique.Three cases with Gartland type III fractures who were initially subjected to closed reduction were later treated by elastic nails subsequent to remanipulations and loss of reduction after closed reduction. Excluding these cases left 74 cases for final review. Each patient's sex and age at the time of trauma were recorded. The type of the fracture was determined according to the modified Gartland classification.^2^,^10^ At the time of presentation, neurovascular complications, associated fractures, and the delay before treatment were also recorded.

The mean age of the 39 boys and 38 girls was 6.3 years (range, 2–11). A total of 42 had the injury on the left side and 35 on the right side. All fractures were treated in 12 h following the accident, except three. Out of 74 children, 4 had preoperative neurapraxias grouped as follows: 3 had median nerve injury and 1 had ulnar nerve injury. All of them recovered with a normal sensory clinical examination. None of the children had preoperative vascular injuries. Two children had ipsilateral torus fractures of the distal radius.

**Figure 1:**
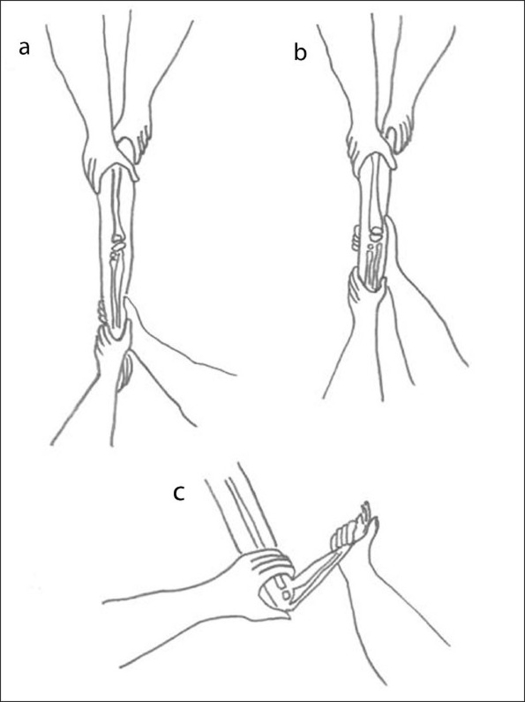
Line diagram showing (a) Traction: axial traction is applied on an extended elbow. (b) Reduction: translation is corrected by collateral pressure with one hand. Rotation is corrected by pronosupination motion of the forearm with the other hand. (c) Stabilization: progressive flexion of the elbow while the thumb pushes the olecranon and the other fingers maintain countertraction at the humeral diaphysis

Three patients with a mild Gartland type II displacement were treated under inhalation of MEOPA (an equimolar mixture of nitrous oxide and oxygen) and intrarectal nalbuphine 0.5 mg/kg. For the 74 other patients, the technique was performed under general anesthesia. The child was positioned at the edge of the operating table with the arm over the image intensifier. Firm, traction was applied with a steady continuous force with the elbow in full extension. Once the deformity in the coronal plane was corrected, the surgeon applied countertraction to the proximal fragment while the thumb reached the olecranon and applied an anterior force to the distal fragment to push back the distal fragment into place [Figure 1]. Concurrently, the other hand flexed the elbow up to 120° and pronated the forearm. When the fracture was reduced, the surgeon evaluated the stability by lateral and medial force applied to the distal part of the humerus. In the case of stability, the reduction was secured by a collar sling fixed to a small cast around the wrist to prevent any loss of forearm pronation. The child was observed overnight to look for any complication. A complete neurological and vascular examination of the involved upper limb was performed before discharge. The immobilization was continued for 3–4 weeks. In case of instability (loss of reduction), the fracture was fixed by an elastic nail and hence was excluded from the study (3 cases).

We analyzed the immediate postreduction radiographs done the next day and radiographs done at the last follow-up: mean 6.5 months (range, 3–25). The measurement of the Baumann angle^11^ was performed on anteroposterior radiographs of the elbow. The measurement of the humerocapitellar angle (shaft-capitelum angle) and lateral rotational percentage^12^ was performed on true lateral radiographs of the elbow in a uniform fashion. Loss of reduction was determined on the basis of the change in the Baumann angle between the immediate postreduction and final follow-up radiographs. According to Skaggs *et al*.,^13^ no displacement was defined as a change in the Baumann angle of <6; mild displacement, as a change of 6–12°; and major displacement, as a change of >12°.

Functional limitation of the elbow range of motion compared with the uninjured side was measured using a goniometer at the most recent office visit: mean 6.5 months (range, 3–25). All patients were also evaluated with the Flynn rating scale at follow-up [Table 1].^14^

**Table 1:** Flynn criteria for grading supracondylar fractures

Rating	Loss of motion (°)	Carrying angle (°)
Excellent	0–5	0–5
Good	5–10	5–10
Fair	10–15	10–15
Poor	>15	>15

To compare continuous data between the groups, the Student test [Table 2] and one-way ANOVA with the post-hoc test were used [Table 3]. The Fisher exact test was used to compare categorical data between the groups in both tables. Statistical analysis was performed with SPSS 15.0 statistical software; *P* values below 0.05 were considered as significant.

**Table 2:** Comparing Gartland II and III fractures

	Gartland II	Gartland III	*P* value
No. of patients	34	40	
Follow-up (months)	4.1	6.8	0.052
Age (year)	6.3 ± 2.2	5.6 ± 2.2	0.070
Sex			0.484
Male	19	18	
Female	15	22	
Neurovascular status			1.000
Median nerve injury	2	1	
Ulnar nerve injury	1	0	
Associated fractures	1	1	1.000
Baumann angle*	73.7 ± 5.2	75.8 ± 5.6	0.078
Humerocapitellar angle*	34.1 ± 8.5	36.8 ± 7.4	0.094
Lateral rotational percentage*	1.9	8.7	0.032
Loss of reduction			0.148
Major	0	3	
Mild	6	11
None	28	26	
Baumann angle**	72.6 ± 3.7	73.8 ± 6.3	0.277
Humerocapitellar angle**	31.7 ± 6.1	40.4 ± 9.4	0.006
Flynn grade**			0.036
Excellent	26	19	
Good	6	10
Fair	2	8	
Poor	0	3	

* Postoperative radiographs,

** Last follow-up.

**Table 3:** Clinical details of Gartland III fractures

	Group A (posterior)	Group B (posteromedial)	Group C (posterolateral)	*P* value
No. of patients	17	12	11	
Age (year)	5.1 ± 2.4	5.5 ± 2.2	6.3 ± 1.9	0.382
Sex				0.848
Male	8	6	4	
Female	9	6	7	
Baumann angle*	75.7 ± 5.3	76.2 ± 5.3	75.4 ± 7.2	0.960
Humerocapitellar angle*	36.9 ± 6.1	34.4 ± 8.9	38.4 ± 7.5	0.440
Lateral rotational percentage*	6.3	1.3	11.6	0.187
Lost of reduction				0.010
Major	0	0	3	
Mild	2	5	4	
None	15	7	4	
Baumann angle**	73.4 ± 4.5	77.7 ± 4.8	68.4 ± 8.4	0.008#
Humerocapitellar angle**	38.3 ± 7.1	35.9 ± 8.2	43.6 ± 13.7	0.351

* Postoperative radiographs,

** Last follow-up,

# Posterior versus posteromedial, *P* = 0.084; posterior versus posterolateral, *P* = 0.048; posteromedial versus posterolateral, *P* = 0.003.

## RESULTS

Thirty-four patients sustained a Gartland type II injury. None of them presented a secondary displacement that required an additional surgical procedure. Treatment results were assessed according to Flynn criteria; the outcome was considered excellent in 26 patients, good in 6, fair in 2, and poor in none. At the last follow-up, all fractures had a normal Baumann angle, except two patients. Mean humerocapitellar angle was 31.7°. One patient presented an ipsilateral wrist fracture and had a fair outcome with a Baumann angle at 86°. The detailed values are given in Table 2.

Forty-three Gartland type III injuries were treated with closed reduction and immobilization with a collar and cuff sling. There were three remanipulations between the first and the eighth postoperative day. Two had cubitus varus deformity and one had cubitus valgus. All three were treated with success by elastic nail fixation and were excluded. Table 3 gives the result for the 40 other fractures that did not require an additional procedure. The preoperative displacement was posterior in 17 patients (group A), posteromedial in 12 patients (group B), and posterolateral in 11 (group C). Treatment results were assessed according to Flynn criteria; the outcome was deemed excellent in 19, good in 10, fair in 5, and poor in 6. All fractures had a normal Baumann angle, except for 7 patients. The mean humerocapitellar angle was 40.4. One patient presented an ipsilateral wrist fracture and has healed without difficulty.

There was no significant difference (*P*>0.05) between the two groups with regard to the average follow-up period, age at the time of fracture, and gender. After closed reduction, there were also no significant differences regarding the Baumann angle and humerocapitellar angle. Only the lateral rotational percentage difference measured after the closed reduction was statistically significant (*P* = 0.032).

Besides the three fractures that needed to be treated by surgery, three other patients had a major loss of reduction [Table 2] in the Gartland type III group and none in the Gartland type II group. Six of the 34 Gartland type II and 11 of the 40 Gartland type III group showed a mild loss of reduction; this was not a significant difference (*P* = 0.148). At the last follow-up there were no significant differences (*P* = 0.277) between the groups regarding the Baumann angle. However, the humerocapitellar angle was 31.7° for the Gartland type II and 40.4° for the Gartland type III group with a significant difference (*P* = 0.006). The Flynn criteria were also significantly different (*P* = 0.036).

Because of the different results between radiographic values and clinical outcomes, we analyzed the Gartland type III fractures in detail [Table 3]. The three groups were similar with respect to demographics and immediate postoperative roentgenograms evaluations. At the last follow-up, the humerocapitellar angles were not significantly different (*P* = 0.351). However, three fractures with posterolateral displacement (group C) had a major loss of reduction whereas none of the other groups showed this. The Baumann angle of group A was 73.4; the angle of group B was 77.7° and of group C was 68.4°. The posterolateral group was significantly different from the other two: A versus C, *P* = 0.048 and B versus C, *P* = 0.003.

One patient experienced an early (before 3 weeks) removal of the immobilization because of elbow eczema. The outcome was fair. None of the patients developed compartment syndrome.

All measurements were done twice by the same two observers (A.G. and D.B.). The measurements were repeated after an interval period of minimum 30 days. The intraobserver error was 1.96° for the Baumann angle and 3.78° for the humerocapitellar angle. The interobserver error was 2.33° for the Baumann angle and 4.40° for the humerocapitellar angle.

## DISCUSSION

There is still a debate regarding the necessity of closed reduction and pinning for all displaced supracondylar fractures, including Gartland type II and type III. Many studies note superior results with closed reduction and pinning.^4^,^5^,^7^,^15^ However, there is some confusion between simple orthopedic treatment by long arm cast^16^ and collar and cuff treatment described by Blount.^9^ Millis *et al*. have shown that failure to flex the elbow over 120° led to a major risk of loss of reduction in cases with a cast immobilization.^6^ Williamson *et al*, reported that approximately 60% of isolated supracondylar fractures could be treated with inelastic strapping and collar and cuff immobilization.^17^ In the English literature, only one study compares the collar cuff immobilization plus a thoracobrachial bandage and the internal fixation, giving no significant advantage to the pin fixation.^18^

Abraham *et al*. demonstrated by experimental hyperextension supracondylar fractures in monkeys that the periosteum tore transversely on the anterior surface of the humerus, at the level of the fracture. Moreover, the periosteal sleeve remains intact on the side with the distal fragment displacement.^19^ This periosteal hinge helps to stabilize the fracture after anatomic reduction. Khare *et al*. showed that the main deforming force is an eccentric compression developed by the triceps tendon when the elbow is flexed to 90° or more.^20^ However, the force applied at the distal end of the radius and ulna by passive pronation of the forearm is transmitted by a see-saw action to counterbalance the deforming force of the triceps.^20^ In fact, when the forearm is in pronation, the compressive forces are concentrated on the center and lateral half of the trochlea and the medial periosteal sleeve is tight.^19^ The reverse findings are noted when the forearm is supinated: compression of the medial joint surface and distraction of the lateral side. The radius and ulna do not cross each other in forearm supination, and no see-saw action occurs. The passive supination force applied at the distal end is directly transmitted proximally giving a lateral distraction. Both studies concluded that the most stable position is full elbow flexion with a 90 forearm pronation.^19^,^20^ In our study, we used a cast around the wrist fixed at the sling to maintain the forearm pronation instead of a collar cuff sling.

Because of the imprecision of the Gartland's original classification, we preferred to use the modified Gartland classification whose reliability has already been proved.^21^ A Gartland type II fracture is displaced (by >2 mm), and the posterior cortex is presumably intact, but hinged.^2^ At the last follow-up, we recorded no major displacement and a minimal change in the Baumann angle. A total of 32 of 34 patients had good and excellent outcomes. No rotational deformity is seen in these fractures because of the intact posterior hinge. While the reduction is complete, a posterior collapse over the bony hinge is probably responsible for the humerocapitellar angle close to 30° instead of 40°. Gartland type III fractures had a humerocapitellar angle closer to the normal value because the posterior hinge is made up of periosteum. They are easy to reduce in the sagittal plane.

**Figure 2:**
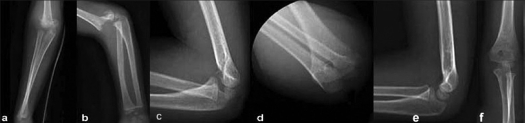
Pre-reduction anteroposterior (a) and lateral (b) radiographs of the elbow of a Gartland type-III fracture with pure posterior displacement. Post-reduction radiographs lateral view (c) and anteroposterior (d) view immobilized by collar cuff and cast around the wrist. Lateral (e) and anteroposterior (f) radiograph of the elbow at eight months follow-up

A Gartland type III fracture is a displaced supracondylar fracture with no meaningful cortical contact.^2^ There is a translational and a rotational displacement which can explain the greater lateral rotational percentage. Because of an early loss of reduction, three patients had to be brought back into the operative room for internal fixation. Even if the final Baumann mean angle is good, only 29 out of the 40 patients had good and excellent outcomes. Table 3 analyzes the preoperative displacement of the Gartland type III fracture. Pure posterior displacement fractures (group A) have an intact posterior periosteal hinge. They are easy to reduce and most of them are stable in full flexion with the forearm in a pronation position [Figure 2]. Laterally torn perioteum is associated with a posteromedially displaced fracture (group B). It is also easy to reduce and is more stable in the same position. The pronation position places the medial periosteum on tension, and lateral compressive force closes the lateral edge to avoid varus malalignment.^19^,^20^ However, a medial periosteum torn is associated with a posterolaterally displaced fracture (group C). Some authors believe that pronation is the most stable position,^19^,^20^ while others think that the pronation position may be counterproductive and suggest the supination position when the lateral periosteum is intact.^2^ If the fracture has no periosteum hinge, it becomes unstable in both flexion and extension. This multidirectional instability is known as Gartland type IV.^22^ These fractures should be treated by internal fixation without any discussion and are not analyzed in this study.

A major loss of reduction is seen for posterolaterally displaced fractures and most of the mild displacement is seen with the posterolaterally and posteromedially displaced fractures. All the fractures were immobilized in pronation except for two posterolaterally displaced fractures that were found more stable in the supination position. The mean value of the Baumann angle at the last follow-up was good for the three groups, but closer to the normal value for the true posterior displacement (group A). Posteromedial displacement (group B) gives a higher Baumann value conducting to cubitus varus deformity. The biomechanical advantage of the forearm prone position^20^ allows avoiding further displacement, especially if the medial periosteum remains intact.^2^ The posterolateral displacement (group C) gives a lower Baumann value conducting to cubitus valgus. In our experience there is no good position to ensure the stability of posterolaterally displaced supracondylar fractures; neither pronation nor supination shows clinical advantage.

Compared to the other methods of stabilization, we had no significant complication: only one early removal of the collar cuff for eczema with a fair functional and radiological outcome. Iatrogenic ulnar nerve injury occurred in less than 5% after pinning a supracondylar fracture in most of the series.^23^–^27^ This high rate of complication is the result of blind pinning and ulnar nerve instability in elbow flexion.^28^ However, lower incidence occurs by using the three lateral pinning technique instead of the cross-pinning^29^ or with a minimal internal approach.^30^ Pin tract inflammation or infection is also an issue.^24^ Fortunately, it usually responds to the removal of the pin and oral antibiotics for a short period. Another advantage of Blount's technique is that for the minimally displaced fractures, general anesthesia is not necessary. In our study, three patients with a mild Gartland type II displacement did not require general anesthesia. They were treated under inhalation of MEOPA and intrarectal nalbuphine, 0.5 mg/kg, with success.

The rate of Volkmann's ischemic contracture (compartment syndrome) in the setting of supracondylar fractures is estimated to be 0.1–0.3%.^2^ Battaglia *et al*. showed that the threshold position for increased forearm pressure is over 90° of elbow flexion.^31^ In this study, like Williamson *et al*.'s study,^17^ we had no case of compartment syndrome. After close reduction, a careful monitoring of the swelling was done for at least 24 h and icing and Non-steroidal anti-inflammatory drugs were used as well. Furthermore, the two cases of supracondylar fractures associated with a wrist fracture were managed without difficulty, while some authors report a higher rate of compartment syndrome with this association.^32^

However, the technique has some limitations. Major elbow swelling compromises a proper and safe elbow hyperflexion. The risk of compartment syndrome is too high and we prefer to use a pinning or elastic nail technique if swelling is too important or if the delay from the trauma exceeds 12 h. A peripheral nerve injury is not a contraindication for closed reduction and collar cuff immobilization as long as the reduction is anatomical and neural examination does not worsen after the reduction. All of our supracondylar fractures with a peripheral nerve injury recovered spontaneously within 10 weeks. However, a vascular injury is a contraindication of Blount's procedure. As mentioned before, nonreducible or unstable fractures after closed reduction cannot be managed by the Blount's technique.

The Blount's technique should not replace pinning. A reducible fracture can be treated by this technique if the fracture is stable under general anesthesia. If not, there is an indication of pinning. If the fracture is not reducible, an open reduction is indicated before pinning the fragments. We recommend the Blount technique with a cast around the wrist to immobilize the forearm in pronation and the elbow in full flexion. Moreover, we recommend a close monitoring of the swelling for at least 24 h. We suggest this technique for all Gartland type II and some Gartland type III fractures. In our opinion, the type III fractures with a posterolateral displacement are less stable and should be treated by internal fixation. According to this study, one-third (74/234) of our supracondylar fractures have been treated by the Blount's technique. Furthermore, the complications associated with systematic pin fixation of all Gartland type II and type III fractures must be weighed carefully against the good outcome achieved using less aggressive and less costly procedures.
